# Supplementary material for: Extracellular Albumin Covalently Sequesters Selenocompounds and Determines Cytotoxicity
Source: Int J Mol Sci. 2019 Sep 24;20(19):4734. doi: 10.3390/ijms20194734 (PMC6801750; doi:10.3390/ijms20194734)
Supplement: Supplementary file 1 [file ijms-20-04734-s001.pdf]

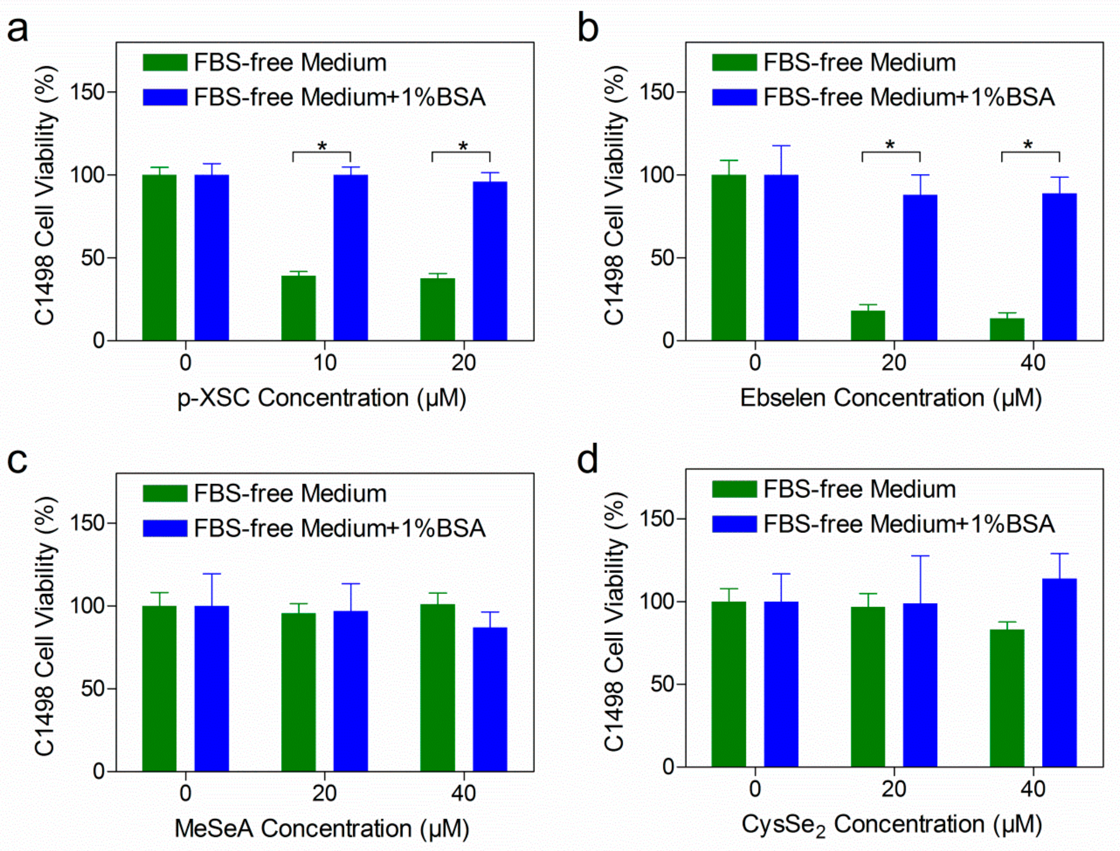

Figure S1. The cytotoxicity of selenocompounds on C1498 cells in FBS-free medium. In all experiments, the treatment duration was 2 hr and cell viability was assayed by CellTiter-Glo kit. The treatment was performed in FBS-free medium without (green bar) or with (blue bar) 1% extra BSA. Results were shown as the mean ± standard deviation of six biological replicates. Two-sided Mann-Whitney test was applied to compare the means between groups. * denotes $p \leqq 0.05$.

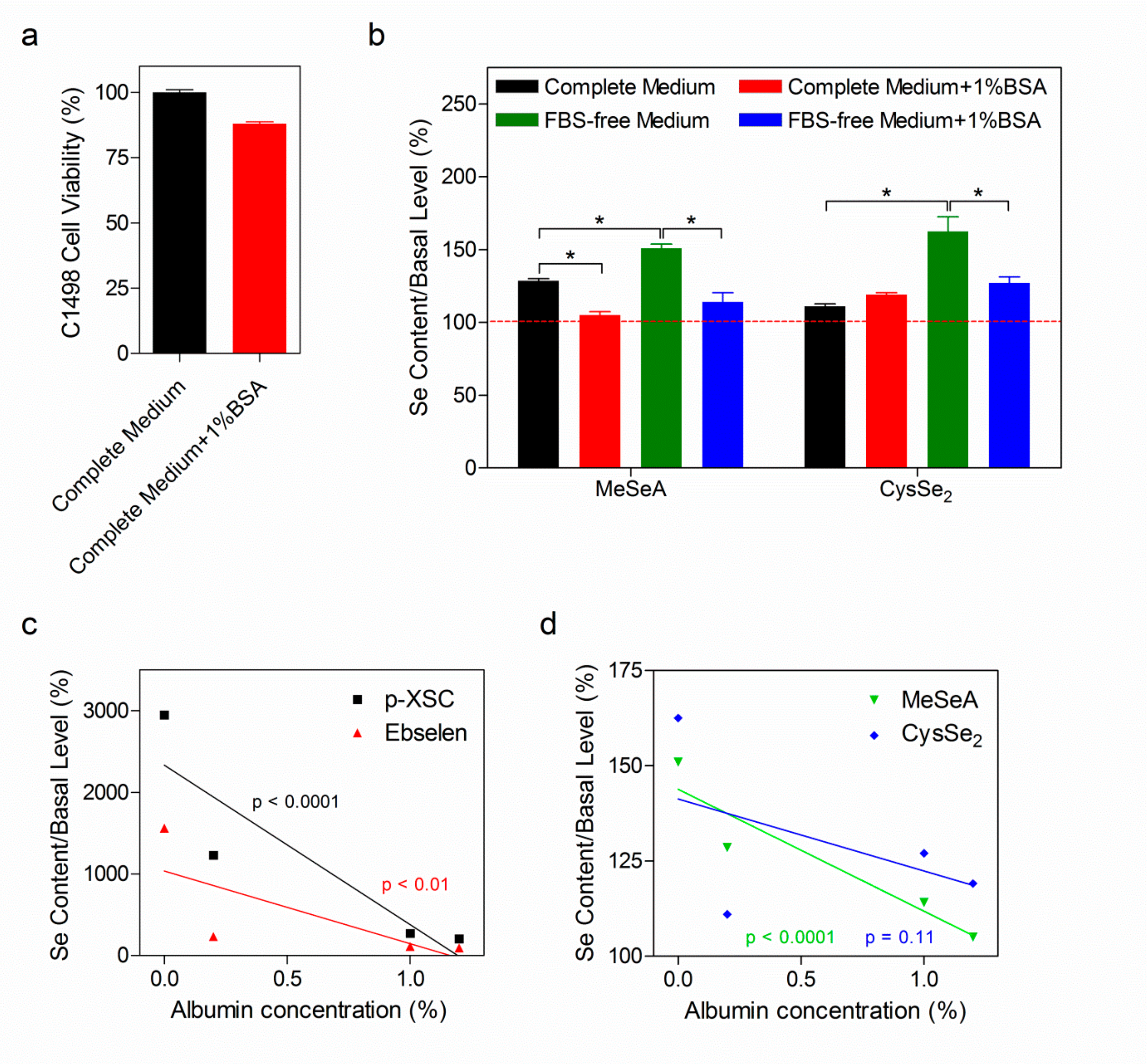

Figure S2. The effect of albumin on cell proliferation and uptake of selenocompounds. (**a**) C1498 cell viability after treatment with 1%BSA for 24 hr (red bar) in relative to untreated cells (black bar). Cell viability was assayed by WST-1 kit and shown as the mean ± standard deviation of six biological replicates. (**b**) Intracellular Se level after MeSeA (20 μM) or $CysSe_2$ (20 μM) treatment for 30 min. The treatment was performed in complete medium (black bar), complete medium supplemented with 1% extra BSA (red bar), FBS-free medium (green bar), or FBS-free medium supplemented with 1% extra BSA (blue bar). Results were shown as the mean ± standard deviation of three technical replicates. The red dash line marks the basal level from untreated cells. Two-sided Mann-Whitney test was applied to compare the means between groups. * denotes $P \leqq 0.05$. (**c-d**) Linear regression between extracellular albumin concentration and intracellular Se level. *p* value indicates whether the slope is significant different to zero. Data were extracted from Figure 2b and Figure S2b.

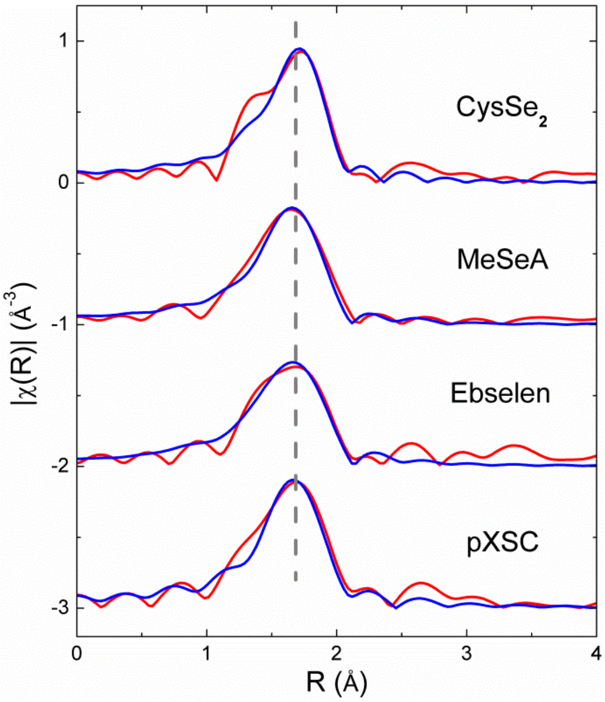

Figure S3. Pseudo-radial distribution function of extended X-ray absorption fine structure. The red and blue lines mark the experimental spectrum and the best fit for SeC-FBS, respectively.

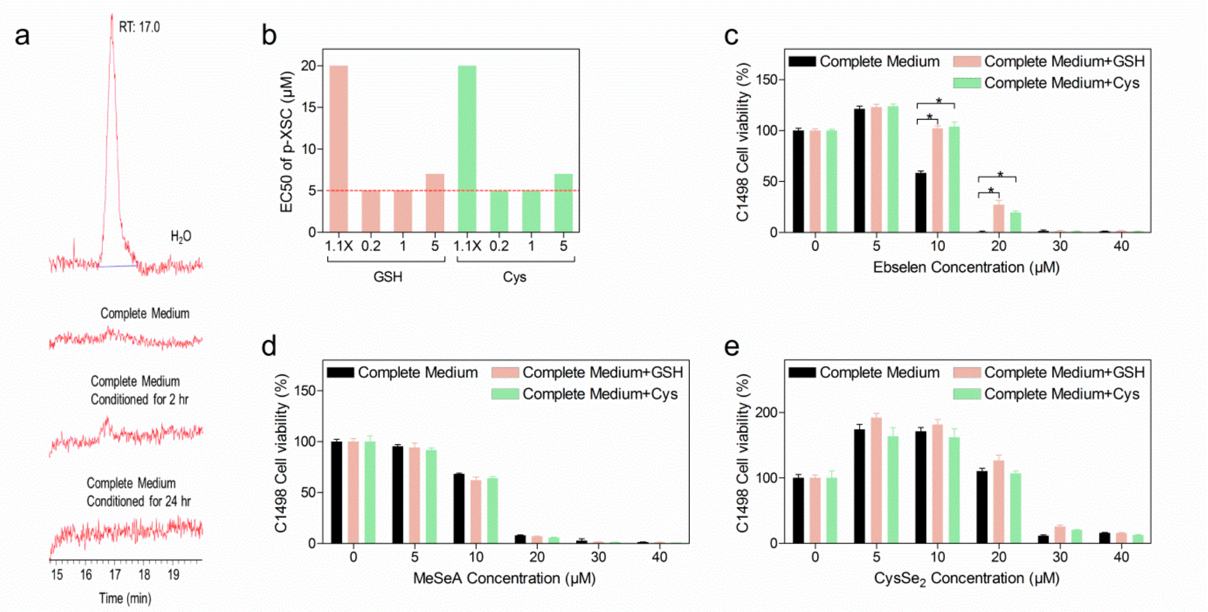

Figure S4. Implication of small molecular thiol in selenocompounds transformation. (a) Chromatograms of p-XSC-SM as acquired from LC-MS analysis. p-XSC was prepared in H_2O, complete medium, or C1498-conditioned (2 hr and 24 hr) complete medium at 10 μM, and then filtered through a centrifugal device with molecular weight cut-off of 10 kDa. p-XSC-SM in the filtrate was quantified thereafter. (b) Effect of GSH and Cys on the EC50 of p-XSC. C1498 cells were concurrently treated with p-XSC and GSH or Cys, and the cell viability was assayed by WST-1 kit 24 hr later. GSH/Cys amount was either in proportion to

p-XSC concentration (1.1-fold higher) or fixed (0.2, 1, and 5 μM). EC50 was defined as the half maximal effective concentration. The red dash line indicates the EC50 of p-XSC alone. (c-e) Effect of GSH and Cys on the cytotoxicity of ebselen (c), MeSeA (d) and $CysSe_2$ (e). C1498 cells were treated with indicated SeC alone or in combination with GSH (10 μM) or Cys (10 μM), and the cell viability was assayed by WST-1 kit 24 hr later. Results were shown as the mean ± standard deviation of six biological replicates. Two-sided Mann-Whitney test was applied to compare the means between groups. * denotes $p \leqq 0.05$.

Table S1. Preparation of SeC-BSA conjugate and SeC-FBS mixture. BSA was dissolved in H_2O at 50 mg/mL.

SeC	SeC-BSA conjugate			SeC-FBS mixture			
	SeC (mg/mL)	SeC (μL)	BSA (mL)	SeC (mg/mL)	SeC (μL)	FBS (mL)	Final (μM)
p-XSC	2	51	2	1.5	84	4	100
Ebselen	2	40	2	1	329	4	300
MeSeA	2	80	2	1	102	4	200
$CysSe_2$	2	80	2	1	401	4	300

Table S2. LC-MS conditions for analysis of free and total p-XSC.

	Item	Free p-XSC	Total p-XSC
LC	Column	YMC AQ12S05-1546WT	
	Mobile Phase	ACN/H_2O with 0.1% Formic Acid	
	Flow Rate (mL/min)	0.2	0.2
	Gradient (%ACN)	70%	70
MS	Spray Voltage (V)	5000	5000
	Sheath Gas (Arb*)	50	40
	Auxiliary Gas (Arb)	5	50
	Ion Sweep Gas (Arb)	2	1.5
	Skimmer Offset (V)	-15	-15
	Capillary Temperature (°C)	375	277
	Tube Lens Offset (V)	100	186
	Ion of Monitor (m/z)	209.9	309.9

Arb*: Arbitrary unit.

Table S3. Fitting results of SeC-BSA spectra considering Se-S bond in the model. The k-range used was 2.5-11.5 Å-1. The amplitude reduction factor (S02) was fixed to 0.85.

SeC	Atom	Number	E_0 (eV)	R (Å)	σ^2 (Å^2)	R-factor
$CysSe_2$	C	1	6.6	2.004	0.003	0.055
	S	1		2.191	0.001	
MeSeA	O	1	10.3	1.980	0.004	0.044
	S	1		2.186	0.005	
Ebselen	C	1	8.1	1.962	0.001	0.032
	S	0.4		2.211	0.003	
p-XSC	C	1	6.2	2.007	0.003	0.062
	S	1		2.184	0.001	
